# An investigation of gene-gene interactions in dose-response studies with Bayesian nonparametrics

**DOI:** 10.1186/s13040-015-0039-3

**Published:** 2015-02-06

**Authors:** Andrew L Beam, Alison A Motsinger-Reif, Jon Doyle

**Affiliations:** 1 Center for Biomedical Informatics, Boston, Massachusetts; 2 Bioinformatics Research Center, North Carolina State University, Raleigh, North Carolina; 3 Department of Statistics, North Carolina State University, Raleigh, North Carolina; 4 Department of Computer Science, North Carolina State University, Raleigh, North Carolina

**Keywords:** Dose-response, Epistasis, Bayesian nonparametric, Neural network, Machine learning

## Abstract

**Background:**

Best practice for statistical methodology in cell-based dose-response studies has yet to be established. We examine the ability of MANOVA to detect trait-associated genetic loci in the presence of gene-gene interactions. We present a novel Bayesian nonparametric method designed to detect such interactions.

**Results:**

MANOVA and the Bayesian nonparametric approach show good ability to detect trait-associated genetic variants under various possible genetic models. It is shown through several sets of analyses that this may be due to marginal effects being present, even if the underlying genetic model does not explicitly contain them.

**Conclusions:**

Understanding how genetic interactions affect drug response continues to be a critical goal. MANOVA and the novel Bayesian framework present a trade-off between computational complexity and model flexibility.

## Background

Understanding the genetic factors underlying differential drug response continues to be a primary goal in pharmecogenomics and drug development. Association studies based on the use of *in vitro* cell lines, such as lymphoblastoid cell lines (LCLs), are becoming an attractive alternative to traditional, human based clinical trials [1-3]. Cell lines offer increased sample sizes relative to traditional studies, resulting in higher statistical power to detect genetic variants that potentially drive drug response. However, the challenges offered by these unique data has yet to be comprehensively studied and evaluated.

Recent work has shown that considering the full set dose-responses instead of summary statistics can greatly increase the power to detect trait-associated genetic variants [3-7]. This new approach is based on a statistical method known as *multivariate analysis of variance* or MANOVA, which is an extension to the well known analysis of variance (ANOVA) framework. Recent studies using a MANOVA-based framework have revealed genetic loci associated with differential responses in anti-cancer agents [3,4]. However, little study to date has been done to investigate the potential effect of gene-gene interactions, or *epistasis*, which is thought to be a critical piece in the genetic architecture of many complex phenotypes [8-10].

The role of epistasis in complex genetic disease continues to be debated in the literature, as well as the potential of epistasis to solve the so-call *missing heritability* problem [11-13]. Recent work has validated the role of epistasis in complex disease in humans [14] and drosophila [15]. To date, nearly all of the work on epistasis has focused on case-control studies, or single-valued QTLs (e.g. gene expression). No work has yet been done to understand how epistasis may affect the unique, multiresponse data that comes from dose-response studies.

To investigate how genetic interactions may affect the power of MANOVA we performed a simulation study across several plausible models of SNP-driven dose-response involving multiple loci. In addition to investigating the MANOVA approach, we develop a novel Bayesian nonparametric model that is capable of automatically accounting for genetic interactions in multi-response data. We have previously developed a Bayesian neural network testing framework [16] for case-control studies that is capable of identifying trait-associated loci in the presence of genetic interactions in a computationally tractable way. In this study, we extend this framework to model dose-response data that contain multiple continuous responses. Through comparisons of several possible genetic models, we hope to gain insight as to how deviation from an additive model (i.e. the presence of epistasis) may affect the statistical power of each method.

## Methods

### Multivariate analysis of variance (MANOVA)

First we provide a brief review of the MANOVA framework for dose-response studies. In a dose-response study, a chemical is administered to a cell culture across several concentrations and a quantitative response (e.g mRNA expression or total ATP) is measured at each concentration. Until recently, the prevailing methodology in dose-response association mapping has been to fit a parametric logistic function, known as the *hill-slope* model, as a function of concentration *c*_*i*_:

(1) $$ f(c_{i}) = Max - \frac{Max - Min}{1+\left(\frac{c_{i}}{{IC}_{50}}\right)^{-w}}  $$

where *Max,Min* are the upper and lower asymptotes, and *w* is the hill-slope. The *IC*_50_ parameter is the concentration at which the response is 50% of maximum and is usually given special importance because it a measure of chemical *potency*. The *IC*_50_ is then treated as quantitative trait and quantitative trait locus (QTL) mapping techniques attempt to identify loci that appear to be associated with this trait. In general, this approach results in a drastic loss of power relative to potential alternatives [7,17]. Recent work has shown that multivariate analysis of variance (MANOVA) has high power across a wide variety of possible dose-response relationships, making it an attractive option for genome-wide association mapping.

Let *x*_*i*_=<*x*_*i*1_,…,*x*_*ip*_>^*T*^ be a vector containing the genotypes of all *p* markers for individual *i*, where each *x*_*ij*_∈{0,1,2} contains the number of instances of the minor allele. Let *y*_*i*_=<*y*_*i*1_,…,*y*_*ik*_> be the vector containing the responses for subject *i* at each of the *k* doses, where each $y_{\textit {ik}} \in \mathbb {R}$. MANOVA models the conditional expected value of *y*, given *x*_*i*_, *E*[*y*_*ik*_|*x*_*i*_] as a linear function:

(2) $$  E[y_{ik}|x_{i}] = \beta_{0} + {\beta_{k}^{T}} x_{i}  $$

The least-squares estimator for *B*_*p*×*k*_=<*β*_1_,…,*β*_*p*_> is given by (*X*^*T*^*X*)^−1^*X*^*T*^*Y*. where *X*_*n*×*p*_=<*x*_*i*_,…,*x*_*n*_>^*T*^ and *Y*_*n*×*k*_=<*y*_1_,…,*y*_*n*_>. For the case when *n*<*p* or when *X* is not full rank, as is often the case for GWASs, smaller sub-models may be fit and analyzed. For example, if 1 million markers are genotyped for only 1,000 individuals, each marker may be fit separately along with any possible confounding covariates such as age, gender, or sub-population status.

This model has several advantages including interpretability and a well understood theoretical foundation that allows for null-hypothesis significance testing. However, the linearity assumption may be somewhat restrictive and if a trait is influenced or determined by interactions between markers, this model may only partially capture the true relationship. One approach to lessen this restriction would be to explicitly include all interactions as terms in the model, and build the linear model on this expanded set of covariates. However, this quickly becomes infeasible for even 2^*nd*^ order interactions. In a study containing one million markers, there are approximately 5∗10^9^ possible 2^*nd*^ order interactions. The computational issues associated with this approach make it infeasible, as do the corresponding multiple testing issues associated with testing billions or trillions of simultaneous hypotheses.

A different approach to account for interactions would be to use a class of models that relax the linearity assumption and impose very little structural constraints on the relationship between *x*_*i*_ and *y*_*ik*_. Neural networks are one such approach and have a rich history of success in the machine learning and genetic epidemiology [18,19]. Neural nets come with several theoretical guarantees that make them appealing for modeling potentially nonlinear functions. Perhaps the most germane property is given by the *universal function approximation* theorem [20], which states that a sufficiently complex neural network is capable of modeling any smooth function on a compact set to an arbitrary degree of precision. This theorem ensures that a neural network is capable in principle of modeling a rich class of functions between input and output. Subsumed in the class of functions neural networks can represent is the linear model used by MANOVA. Thus, if the true model is indeed linear, the relationship learned by the neural network would automatically collapse to represent the linear mapping between input and output, while if the true relationship is more complex, it will include any nonlinearities without having to specify them *a priori*. In the next section we present and develop a Bayesian neural network framework for dose-response studies.

### Bayesian neural networks

Bayesian neural networks represent an extension to the familiar neural network framework. Recent work in [16] has shown they can successfully model genetic interactions in case-control studies. Here we present an augmented design capable of modeling multi-response data, such as the kind observed in dose-response studies.

**Figure 1:**
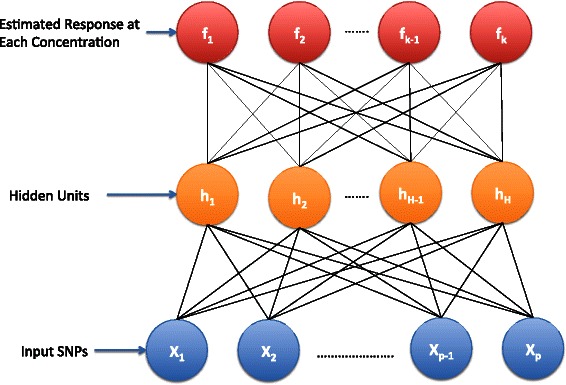
**Graphical depiction of a neural network with several output units.** The blue nodes on the bottom represent SNPs in MAF coding, the orange nodes represent the hidden unit functions in Equation (3), and the red nodes at the top represent the estimated response for each of the concentrations measured. This architecture allows the network to model the response at each concentration as a nonlinear combination of the input SNPs.

Neural networks construct a hierarchical representation where inputs are transformed into (potentially) nonlinear features that can undergo further nonlinear transformations. For our purposes we consider networks with only one such transformation. Networks with this architecture are often described as having one *hidden layer*, because the graphical model describing the network has one such transformation occurring at the same hierarchical level between input and output. Figure 1 shows a graphical depiction of a neural network model for dose-response data.

The first layer of Figure 1 shows the input SNPs represented as the number of minor alleles present at each of the *p* markers. The inputs are fed into several *hidden units*, where each hidden unit, *h*_*j*_(*x*_*i*_), performs the following nonlinear *logistic* transformation:

(3) $$  h_{j}(x_{i}) = \frac{1}{1+\exp\left(-\left(a_{j} + {x_{i}^{T}}w_{j}\right)\right)}  $$

where *w*_*j*_ is a *p*×1 vector of weights associated with hidden unit *j*, and *a*_*j*_ is the unit’s bias. The outputs from the hidden units are then fed into each output unit (*f*_*k*_(*x*_*i*_)). Each output unit takes a linear combination of the outputs from the hidden units, using the following form:

(4) $$  f_{k}(x_{i}) = \alpha_{k} + \sum\limits_{j=1}^{H} h_{j}(x_{i})*\beta_{kj}  $$

where *α*_*k*_ is the bias and *β*_*kj*_ is the weight given to the output from hidden unit *j*, for output unit *k*. Equations (3) and (4) form the base neural network model. To incorporate this into a Bayesian framework, we must define several additional components. Bayesian methods define a posterior distribution over possible parameter values. The posterior distribution combines a prior distribution, that is updated based on the observed data. Letting *θ* represent all of the network parameters in Equations (3), (4), Bayes’ rule defines the posterior distribution of *θ*, given the data *x*_*i*_, *y*_*i*_:

(5) $$  p\left(\theta| x_{i},y_{i}\right) = \frac{L\left(\theta|x_{i},y_{i}\right) p(\theta)}{\int L\left(\theta|x_{i},y_{i}\right)p(\theta) d\theta}  $$

where *p*(*θ*) is the prior distribution for the network parameters and *L*(*θ*|*x*_*i*_,*y*_*i*_) is the likelihood function. The network’s output for each observation, *f*_*ik*_(*x*_*i*_), is given an independent Gaussian likelihood with mean *f*_*ik*_(*x*_*i*_) and unit variance, shown below:

(6) $$ L\left(\theta |x_{i},y_{i}\right) = \exp\left(-\frac{(y_{ik} - f_{ik}(x_{i}))^{2}}{2} \right)  $$

There are many choices for the prior, *p*(*θ*), but the one adopted here is the *Automatic Relevance Determination* (ARD) prior [21,22]. Briefly, the ARD prior groups weights in the hidden layer together in a meaningful way. Weights that are connected to the same SNP across hidden units are given a shared parameter that controls how large the values they take on are allowed to become. Specifically, the weights in each hidden unit that are associated with SNP *j* are given the following hierarchical prior:

(7) $$ \beta_{kj} \sim N\left(0,{\sigma_{j}^{2}}\right)  $$

(8) $$ \sigma_{j}^{2} \sim IG\left(\alpha_{0},\beta_{0}\right)  $$

where *N*(·,·) and *IG*(·,·) represent Normal and Inverse-Gamma densities, respectively. This prior allows the network to automatically determine which inputs are the most relevant. If SNP *j* seems to be related to the response, then ${\sigma _{j}^{2}}$ will be concentrated around large values in the posterior. We have previously developed a Bayesian framework that allows for testing of variable importance relative to a null model [16]. This framework provides a posterior probability of each SNP’s involvement in the response and is capable of being applied to continuous, dose-response data in addition to the case-control scenario for which it was originally developed.

**Figure 2:**
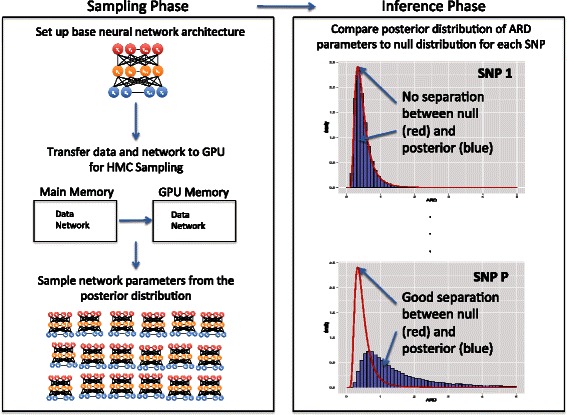
**Overview of the Bayesian neural network method for dose-response studies.** First the network architecture is established and transferred along with the data to GPU memory. The HMC simulation is performed on the GPU and the samples are then transferred back to main memory. The posterior for each SNP’s ARD parameter is compared to the null distribution. Bayesian posterior probabilities are computed to assess how likely each SNP is to be involved in determing drug-response. In the right panel SNP 1 shows little evidence of being involved with this trait while SNP P has strong evidence of playing a role.

The denominator of (5) is an integral that, in high-dimensions, is intractable. Thus stochastic integration techniques, such as Markov Chain Monte Carlo (MCMC) are often used. MCMC algorithms are able to draw samples from the posterior distribution *p*(*θ*|*x*_*i*_,*y*_*i*_) and only require the ability to evaluate *L*(*θ*|*x*_*i*_,*y*_*i*_)*p*(*θ*) for specific values of *θ*. However, popular algorithms such as the well known random-walk Metropolis-Hastings (RW-MH) [23,24] explore the posterior distribution too slowly to be useful in a high-dimensional setting. Recently an algorithm known as Hamiltonian Monte Carlo (HMC) has been shown to explore high-dimensional posterior distributions much more efficiently by leveraging information about the posterior’s gradient, which guides the simulation to regions of high probability. This efficiency does not come free, as evaluation of the gradient must be done 10s to 1000s of times *per iteration*, possibility limiting HMC’s usefulness when the number of parameters is large. To enable the use of HMC on complex models in high-dimensions, we have developed a GPU-based sampling scheme that allows neural network models to be used on large datasets [25]. A graphical overview of the entire Bayesian neural network procedure is provided in Figure 2.

This framework comes with several advantages relative to the MANOVA approach. As discussed previously, the relaxation of the linear assumption allows for a broader class of possible genetic architectures to be detected. Additionally, the BNN approach provides a nonparameteric procedure for variable selection while MANOVA relies on normality of errors for its testing procedure to be valid. For instance, p-values from a MANOVA analysis may not be valid for scenarios in which the number of individuals for one genotype is rare (e.g. very few homozygotes for the minor allele). Additionally, normalization procedures must often be performed in order to ensure the error distributions conform as closely as possible to the assumed distribution. In contrast, due the lack of strict assumptions required by the Bayesian neural network framework, there is no reason in principle why a BNN would be affected by any of these issues.

## Results and discussion

We investigated three plausible relationships between genotype and drug-response. For all simulations a Bayesian neural network with 10 hidden units was used. The HMC simulation was performed for 375 iterations, with the first 25 discarded as burn-in. For the HMC sampler a step-size value of 0.02, a momentum-persistence value of 0.75, and a temperature value of 1000 were used. The ARD hyper-parameter values were set to *α*_0_=3 and *β*_0_=1 while the output layer used *α*_0_=0.1 and *β*_0_=0.1, and we used an ARD cut-off value of 0.4. The ARD hyper-parameters, *α*_0_, *β*_0_ control the shape and scale (respectively) of the inverse-gamma prior distribution in the ARD hierarchy. This prior distribution controls the degree to which weights in the hidden layer are constrained, relative to a standard neural network. In our experience, the network is relatively insensitive to these values but please see [16] for the explicit role these parameters play. Each response was normalized to have mean 0, unit variance. All BNN code was written in Python and is avilable at https://github.com/beamandrew/BNN.

We used a Bonferonni cut-off value of *p*<0.05 for the MANOVA procedure. MANOVA was conducted using the manova() function in R. Processing each file file took the BNN approximately 1 minute, while the MANOVA procedure took approximately 5 seconds for all simulation settings.

### Additive model

As a baseline for the Bayesian neural network model, we first performed a simulation study using a simple additive model involving 2 loci. We generated a mean dose-response for each concentration, *μ*=<*μ*_1_,…,*μ*_6_>, according to the hill-slope model in Equation (1):

(9) $$ \mu_{k} = 1 - \frac{1}{1+x_{k}^{-1.5}}  $$

for *k*∈{1,…6} and *x*=10^−4^∗{0.03125,0.0625,0.10,0.25,0.5,1.25}, resulting in a mean dose-response curve for 6 concentrations. Deviations from the mean induced by SNP status were generated according to a linear model plus a heteroskedastic noise term, yielding an observation *i* at each concentration, *y*_*ik*_:

(10) $$ y_{ik} = \mu_{k}*\left(1 + \frac{\theta}{2}*S_{1} - \frac{\theta}{2}*S_{2} + \epsilon \right)  $$

where *ε*∼*N*(0,0.1) and *S*_1_,*S*_2_∈{0,1,2} represent number of minor alleles at the two causal loci, and *θ*∈[0,1] represents the effect size of each SNP. In this model being homozygous for the minor allele causes a *θ*% change relative to the baseline mean, *μ*_*k*_. For example, *θ*=0.02 would correspond to a 2% change for a subject that is homozygous for the minor allele at *S*_1_ while being homozygous for the major allele at *S*_2_:

$$y_{k} = \mu_{k}*\left(1 + \frac{0.02}{2}*2 - \frac{0.02}{2}*0 +\epsilon \right) $$

$$= \mu_{k} + \mu_{k}*0.02 + \epsilon_{k} $$

**Figure 3:**
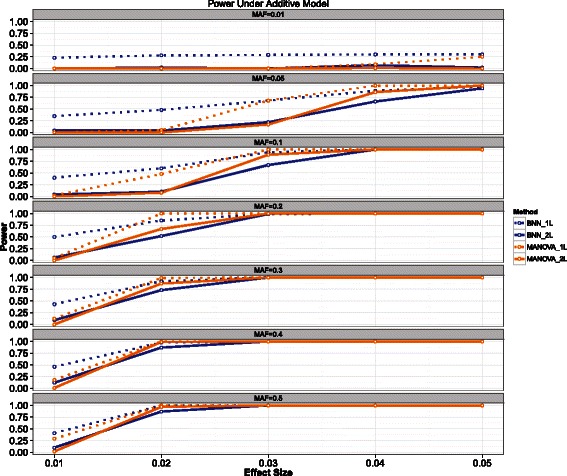
**Power results for the additive model.** The power for the Bayesian neural network (BNN) model is shown in blue while MANOVA is shown in orange. Solid lines indicate the power to detect both loci, while the dashed lines indicate power to detect at least one of the causal loci.

Also note that one locus (*S*_1_) is associated with increased drug response while the other locus (*S*_2_) confers a decreased average response. To evaluate the sensitivity of both MANOVA and BNN to effect size (*θ*) and minor allele frequency (MAF), we simulated data sets for *θ*={0.01.0.02,0.03,0.04,0.05} and MAF ={0.01,0.05,0.1,0.2,0.3,0.4,0.5}. For each combination of *θ* and MAF, we generated 100 data sets resulting in 3500 data sets used in evaluation. Each dataset contained 2,000 observations and 998 background SNPs. Background SNPs were generated according to a random MAF, ranging uniformly from 0.01 to 0.5. For each parameter combination, the number of times out of 100 each method correctly identified both causal SNPs as an estimate of statistical power. The results are shown in Figure 3.

Both BNNs and MANOVA display good power across a variety of parameter combinations, with MANOVA having a slight edge for a few parameter combinations. This is expected as MANOVA is testing the linear hypothesis directly, while the BNN is testing a much more general hypothesis.

### Additive model with interactions

Next, we considered an additive model with an interaction term, shown below.

(11) $$ y_{k} = \mu_{k}*\left(1 + \frac{\theta}{2}*S_{1} - \frac{\theta}{2}*S_{2} + \frac{\theta}{2}*S_{1}*S_{2} +\epsilon\right)  $$

**Figure 4:**
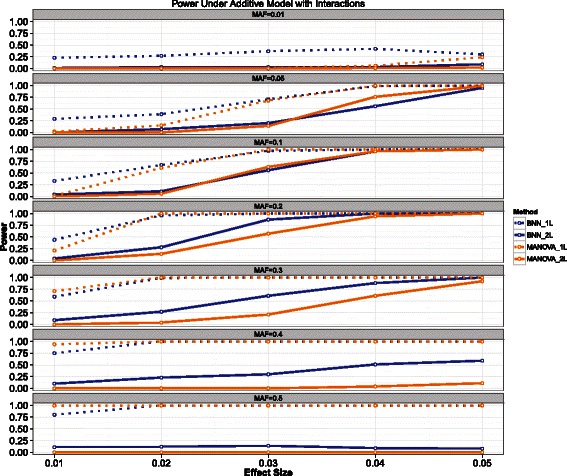
**Power results for the additive model with interactions.** The power for the Bayesian neural network (BNN) model is shown in blue while MANOVA is shown in orange. Solid lines indicate the power to detect both loci, while the dashed lines indicate power to detect at least one of the causal loci.

In this model there is deviation from additivity induced by the the interaction term *S*_1_∗*S*_2_. We again swept over the same range of values for *θ* and MAF as done previously. The results are shown in Figure 4.

Again, both methods appear to have good power across a wide spectrum of parameter values. However, MANOVA does not perform as well as for larger values of MAF. The BNN also experiences a loss of power for the highest level of MAF. For most parameter combinations, the BNN outperformed MANOVA.

### Purely interactive model

**Figure 5:**
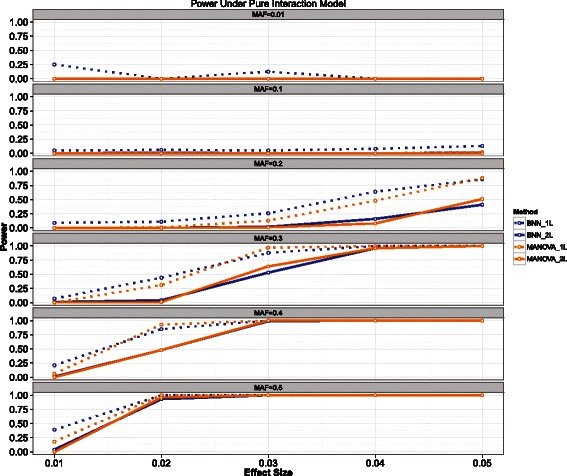
**Power results for the purely interactive mode.** The power for the Bayesian neural network (BNN) model is shown in blue while MANOVA is shown in orange. Solid lines indicate the power to detect both loci, while the dashed lines indicate power to detect at least one of the causal loci.

Finally, we investigated a model in which the SNPs affect the response *only* through an interaction, shown below. The results are shown in Figure 5.

(12) $$ y_{k} = \mu_{k}*\left(1 + \frac{\theta}{2}*S_{1}*S_{2} +\epsilon\right)  $$

Perhaps surprisingly, MANOVA displays good power to detect the causal loci for several parameter combinations, despite the true model ostensibly lacking any marginal effects. Both methods struggled for smaller values of MAF, but this is expected due the relatively rare nature of positive responses for small levels of MAF.

### Further analysis of simulated models

The results of the preceding section motivated a more in-depth analysis of the reasons behind MANOVA’s apparent ability to detect models that only contain interactions and the reduced ability by both methods to detect the model containing main effects and an interaction term. The apparent loss of power by both methods for higher values of MAF in Figure 4 may be an artifact of the specific simulation parameters used. Notice for example, that both MANOVA and BNNs have good power to detect one locus for all simulated scenarios, as evidenced by the dotted line. This suggests that the loss of power under this model is due to the decreased ability to detect the second locus (*S*_2_), as examination of the raw simulation results indicates this locus was rarely identified as significant. To examine this hypothesis, we will assume a more general population genetics framework and then reexamine the results of the simulated models in the previous section as specific instances of this broader viewpoint.

A marginal effect is the expected value of *y*_*ik*_ given the status at one locus, averaged over all possible values for all other loci. Since the MANOVA procedure only detects marginal effects, a deeper understanding of how they may manifest in these putative models of genetic influence may be valuable. What we intend to show is that the discrete nature of the minor allele coding can introduce artifacts such as main effects in models where none are explicitly present and how marginal effects are attenuated by an interaction term in models containing both. First, we will examine the marginal effect of having a specified genotype at one locus (e.g. *S*_2_=1) independently of the status at the other locus, and determine generally how this marginal effect changes under different simulated models as a function of effect size and MAF.

For the moment assume we an infinite population of individuals and we are interested in the relationship between two loci *S*_1_ and *S*_2_ and a quantitative trait, *y*. Let *p*_*xy*_ be the proportion of individuals containing *x* copies of the minor allele for locus *S*_1_ and *y* copies of the minor allele for locus *S*_2_. As done previously in Equation (11), assume that the expected value of *y*, *E*[*y*] is given by an additive relationship:

(13) $$  E[y] = \beta_{1}*S_{1} + \beta_{2}*S_{2}  $$

where *β*_1_,*β*_2_ are the effects of having the *S*_1_,*S*_2_ genotypes, respectively. Consider the marginal effect of having the genotype *S*_2_=1. The conditional expectation of y given *S*_2_=1, *E*(*y*|*S*_2_=1), can be computed using the law of total probability, with a weighted sum over all possible values for *S*_1_:

(14) $$ E\left[y|S_{2} = 1\right] = \sum\limits_{i=0}^{2} E\left(y|S_{2}=1,S_{1}=i\right) * p_{i1}   $$

Substituting Equation (13) into (14) and simplifying:

$$\begin{aligned} E\left[y|S_{2} = 1\right] &= E\left[y|S_{2}=1,S_{1}=0\right] \cdot p_{01} + E\left[y|S_{2}=1,S_{1}=1\right] \cdot p_{11}\\ &\quad+E\left[y|S_{2}=1,S_{2}=2\right] \cdot p_{21}\\ &= \beta_{2} \cdot p_{01} + \left(\beta_{1} + \beta_{2}\right) \cdot p_{11} + \left(2\beta_{1} + \beta_{2}\right) \cdot p_{21} \end{aligned} $$

Rearranging and simplifying the last line yields:

(15) $$ E\left[y|S_{2} = 1\right] = \beta_{1}\cdot \left(p_{11} + 2p_{21}\right) + \beta_{2} \cdot \left(p_{01} + p_{11} + p_{21}\right)  $$

Let the conditional expectation shown in Equation 15 be referred to as the marginal effect of *S*_2_=1 under the additive model, or *μ*_*A*_. Now consider that instead of following a simple additive model, the expected value of *y* is given by an additive model plus an interaction term, shown below:

(16) $$ E[y] = \beta_{1}*S_{1} + \beta_{2}*S_{2} + \beta_{3}*\left(S_{1} \cdot S_{2}\right)  $$

Again, using a similar approach as before and summing over all possibilities for *S*_1_ the conditional expected value is:

(17) $$ E\left[y|S_{2} = 1\right] = \beta_{1}\left(p_{11} + p_{21}\right) + \beta_{2}\left(p_{01} + p_{11} + p_{21}\right) + \beta_{3}\left(p_{11} + 2p_{21}\right)  $$

Let this expected value be referred to as *μ*_*AI*_. Equations (15),(17) give the marginal effect of having the genotype *S*_1_=1 under an additive model and an additive model with an interaction for arbitrary effect sizes and for arbitrary genotype frequencies. Note that if *β*_1_,*β*_2_,*β*_3_>0, then *μ*_*AI*_>*μ*_*A*_ or the marginal effect under the model with interactions would be larger, thus MANOVA would have higher power to detect this SNP as causal. However, using the values used in the simulation ($\beta _{1} = \beta _{3} = \frac {\theta }{2}, \beta _{2} = -\frac {\theta }{2}$) the equations reduce to the following expressions:

$$\begin{aligned} \mu_{A} &= -\frac{\theta}{2}(p_{01} - p_{21}) \\ \mu_{AI} &= -\frac{\theta}{2}(p_{01} - p_{11} - 2p_{21}) \end{aligned} $$

Here it is clear that |*μ*_*AI*_|<|*μ*_*A*_| since *p*_*xy*_>0∀*x*,*y*. This is due to the negative *S*_2_ effect, which induces a *smaller* marginal effect for this locus under the additive model with an interaction. This explains why in the simulations the power to detect this locus was reduced when compared to the purely additive model. The reason MANOVA has reduced power when MAF is high because the interaction term appears more often and since it has the opposite sign as the *S*_2_ locus, it effectively cancels out the response, making it appear as if *S*_2_ has a smaller marginal effect. Table 1 shows the values for *μ*_*AI*_ and *μ*_*A*_ for each possible level of *S*_2_ for the effect sizes used in the simulations (i.e. $\beta _{1} = \beta _{3} = \frac {\theta }{2}, \beta _{2} = -\frac {\theta }{2}$):

**Table 1:** **Relationship beteween** ***μ***_***AI***_ **and** ***μ***_***A***_ **for each possible level of** ***S***_**2**_

*S*_2_Status	*μ*_*AI*_	*μ*_*A*_	|*μ*_*AI*_| ? |*μ*_*A*_|
0	$\frac {\theta }{2}(p_{10} + 2p_{20})$	$\frac {\theta }{2}(p_{10} + 2p_{20})$	=
1	$-\frac {\theta }{2}(p_{01} - p_{11} - 2p_{21})$	$-\frac {\theta }{2}(p_{01} - p_{21})$	<
2	$-\frac {\theta }{2}(2p_{02} - p_{12} + 2p_{22})$	$-\frac {\theta }{2}(2p_{02} + p_{12})$	<

Note that all of the marginal effects for *S*_2_ are smaller than or equal to the additive models marginal effect size. This explains why there was a decrease in power to detect *S*_2_ by MANOVA in the simulations.

Now we turn to the model without any explicit marginal effects and examine if there are indeed any marginal effects present. Recall the purely interactive model is *E*[*y*]=*S*_1_∗*S*_2_∗*β*_3_. Using the same analysis procedure as before we can calculate the marginal effect under an interactive model, *μ*_*I*_ for each level of *S*_2_. This is shown in Table 2:

**Table 2:** **Relationship beteween** ***μ***_***I***_ **and** ***μ***_***A***_ **for each possible level of** ***S***_**2**_

*S*_2_Status	*μ*_*I*_	*μ*_*A*_	|*μ*_*I*_| ?|*μ*_*A*_|
0	0	$\frac {\theta }{2}(p_{10} + 2p_{20})$	<
1	$\frac {\theta }{2}(p_{11} + 2 p_{21})$	$-\frac {\theta }{2}(p_{01} - p_{21})$	?
2	$\frac {\theta }{2}(p_{12} + 4p_{22})$	$-\frac {\theta }{2}(2p_{02} + p_{12})$	?

A ? in the third column indicates that no strict inequality can be determined.

Here there is no clear, strict inequality, but assessments can be made for specific values of *p*_01_,*p*_11_,*p*_21_. Note that in general |*μ*_*I*_|>0 in all instances so there will *always* be some amount of marginal effect present for this model. For many values of MAF, |*μ*_*I*_| will be a non-trivial amount relative to |*μ*_*A*_| resulting in high power for a model can only detect additive effects, such as MANOVA. Thus the reason MANOVA in Figure 5 has good power for high values of MAF is because there actually are marginal effects present, even though they were not explicitly included in the model.

The analysis in this section is meant as a small complement to the vast literature on quantitative trait loci (QTL) and the role of epistasis in settings other than the dose-response framework. Several comprehensive investigations have been made using model organisms such as *Drosophila melanogaster* [15,26] for which there is considerable evidence of the role of epistasis in quantitative traits. How the results of these simple, 2 loci models might translate to larger epistatic networks in other contexts involving many more loci is yet unclear. However, the simulated models in this study suggest that epistatic interactions in a dose-response framework may manifest as marginal effects for each loci involved in the interaction, at least for some configurations of the minor allele frequency.

### Analysis of the anticancer agent etoposide

In this section we apply the the Bayesian neural network to real dose-response data originally presented in [3]. We focus on the anticancer agent Etoposide which is a topoisomerase inhibitor used in the treatment of wide variety of cancers. Etoposide was chosen for analysis due to the high heritability of cytotoxicity for this compound (approximately 40% heritable) [27] obvserved in previous studies, yet estimated marginal effects have only been able to account for a small fraction of this heritability.

The cytotoxicity of Eptoposide at six concentrations was assessed via cell viability counts in lymphoblastoid cell lines (LCLs) of 520 individuals of European descent. Genotype status for each individual was determined for either 314,621 or 620,901 SNPs, using HumanHap300 bead chip or HumanQuad610 bead chip platforms [3]. Status for 2.5 million total SNPs was imputed using the approach in [28]. Quality control on the 2.5 million SNPs was performed as in [3]. SNPs failing a test for Hardy-Weinberg equilibrium at the *p*=0.01 level were removed, which resulted in approximately 30,000 SNPs being discarded. QC and initial data preparation were performed using the software package plink [29]. In the previous section it was shown that even SNPs contributing to genetic risk through an interaction term only will still most likely result in observed marginal effect. Using this rationale we first screened for marginal SNP effects using MAGWAS [17]. Following [3], we included temperature, growth rate, the first 3 principal components, and the experimental batch as covariates in the model. After screening for SNPs with statistically significant marginal effects at the *p*<10^−5^ level, we were left with 41 SNPs for analysis by the Bayesian neural network. Genome-wide screening using MAGWAS took approximately an hour on an Intel i5, quad-core desktop CPU.

We fit a neural network model with 10 hidden units, a logistic activation function and six Gaussian output units (one for each concentration). Sampling was done for 25 burn-in iterations, followed by 2,000 iterations to be used for inference. The sampler settings were as follows: a of step-size 4∗10^−2^, *L*=20 leap-frog steps per iteration, and an initial annealing temperature of *T*_0_=1000. The ARD prior parameters were set to *α*_0_=5,*β*_0_=2. Please see [16] for more detail and discussion on these parameter settings. Sampling took approximately 10 minutes on a GeForce GTX 650 desktop GPU. The HMC analysis procedure was performed for 3 independent replications and trace plots for each variable were inspected to assess if parameter estimates had converged.

The top SNP from [3] (rs2076112) has a Bayesian posterior probability which ranks it as the 20th most important SNP of the 41 SNPs passing the MAGWAS filter. As was found in [3], several experimental condition variables were found to be very important by the BNN as well. The top 7 most important variables were all related to experimental and cellular growth conditions, underscoring the importance of accounting for these conditions as covariates when performing cell-based gene-mapping. The top SNP according the BNN was rs12650820 (Bayesian probability 0.20, MAGWAS p-value 7.878∗10^−6^), a SNP located on chromosome 4 within the follistatin-like 5 (FSTL5, location 4q32.3) gene. Dysfunction of FSTL5 has been implicated as biomarker in medulloblastoma, a highly malignant primary brain cancer [30]. All of the remaining SNPs have marginal importance scores which are suggestive of involvement, but less definitive.

## Conclusions

In this study we have examined how gene-gene interactions can affect the ability to detect trait-associated loci in cell-based, dose-response association studies. We have presented a novel nonparametric procedure in the form of a Bayesian neural network and compared its performance to the MANOVA framework. Using a simulation study of plausible genetic models and a population genetics based analysis, we have shown that MANOVA may be able to detect causal loci even in the presence of genetic interactions. Additionally, we have shown that the BNN is also very capable of detecting causal loci across a range of possible genetic models. Each approach comes with trade-offs - the MANOVA approach is conceptually more simple and computationally less expensive while the BNN approach is more flexible and built upon fewer assumptions. In light of the results of this study, we recommend a two-stage approach (as was used in the Etoposide analysis) as a viable analysis strategy. MAGWAS is able to screen millions of SNPs quickly for marginal effects, which will mostly likely be present even if the underlying genetic architecture is epistatic. Next, the Bayesian neural network can be employed on a smaller subset of SNPs to explore a richer model space.

It’s also worth discussing some of the modelling benefits offered by the Bayesian neural network approach. Due to flexibility afforded by the BNN framework, new constraints or data types can be easily accommodated. Note that to model a new type of data, one only must be able to write down the likelihood and incorporate this into the output layer of the network. Thus, when analysing a new type of data, the only change that must be made is the type of output layer used, which reflects the new data’s likelihood function. Everything else, such as input layer, hidden layers, and ARD-testing framework, remain the same. There is no assumed model and no distributional assumptions, making this proposed framework very widely applicable to a variety of association mapping scenarios. More work remains to be done, however, before this approach can be used in a more turn-key manner. The most immediate improvement that can be made is to the HMC-based sampler. As with any MCMC method, sub-optimal parameter values will result in a chain failing to converge. The HMC-MCMC sampler used here is no exception and requires several tuning parameters that can dramatically impact the performance of the approach as a whole. Most often, several pilot runs are performed to find good values for these parameters. Recently, several groups have improved upon the generic HMC sampling scheme we used [31-33] which alleviate some of these issues. Incorporating these recent advances in HMC sampling techniques will help to make the method more robust and user-friendly.

In this study we have investigated how genetic interactions may affect the MANOVA analysis procedure, provided a deeper understanding of how these interactions may affect dose-response data, and presented a novel Bayesian nonparametric analysis technique. Cell-based dose-response studies hold much promise for pharmacogenomics, and the work presented here will help future research efforts utilizing this type of data.

## Software availability

Software implementing the approaches outlined in this paper is available at https://github.com/beamandrew/BNN.
